# Effect of fenofibrate on uric acid level in patients with gout

**DOI:** 10.1038/s41598-018-35175-z

**Published:** 2018-11-13

**Authors:** Ju-Yang Jung, Young Choi, Chang-Hee Suh, Dukyong Yoon, Hyoun-Ah Kim

**Affiliations:** 1 Department of Rheumatology, Ajou University School of Medicine, 164 Worldcup-ro, Yeongtong-gu, Suwon, 16499 Korea; 2 Department of Biomedical Informatics, Ajou University School of Medicine, 164 Worldcup-ro, Yeongtong-gu, Suwon, 16499 Korea; 3 Department of Biomedical Science, Ajou University School of Medicine, 164 Worldcup-ro, Yeongtong-gu, Suwon, 16499 Korea

## Abstract

Gout is a chronic disease associated with deposition of monosodium urate crystals and accompanied by diabetes, hypertension, and dyslipidemia. Hypertriglyceridemia is common among patients with gout, and fenofibrate is usually used to reduce triglyceride levels. The aim of this study is to determine the effect of uric acid reduction by fenofibrate in patients with gout administered uric acid lowering agents (viz., the xanthine oxidase inhibitors allopurinol and febuxostat). Data from 863 patients with gout were collected from electronic medical records comprising information on underlying diseases, laboratory findings, and drug histories. Among all the patients, 70 (8.11%) took fenofibrate with allopurinol or febuxostat. Male and young patients took fenofibrate more frequently, and hypertension was less frequent in patients administered xanthine oxidase inhibitors and fenofibrate than in those administered only xanthine oxidase inhibitors. After the treatment, serum uric acid levels more significantly decreased (−1.81 ± 2.41 vs. −2.40 ± 2.28 mg/dL, p = 0.043) in patients with fenofibrate cotreatment, than in those administered allopurinol or febuxostat alone. The effect of uric acid reduction was larger (b = −1.098, p < 0.001) in patients taking glucocorticoids than in those administered other treatments. There was no difference in the levels of creatinine, blood urea nitrogen, and aminotransferases between patients treated with and without fenofibrate. Fenofibrate additionally reduced uric acid levels without showing any change in the results of renal or liver function tests, suggesting that the addition of fenofibrate is a reasonable option for treating gout in patients having high triglyceride levels.

## Introduction

Gout is a chronic disease caused by inflammatory responses to the deposition of monosodium urate (MSU) crystals. Elevated serum urate concentration leads to the crystallization of MSU, and hyperuricemia is an important etiologic factor in the development of gout^1,2^. Uric acid is the final product of metabolism of endogenous and ingested purine in humans^3^. Increased production or inadequate renal excretion of uric acid is the main cause of hyperuricemia, while diet and comorbidities including obesity and renal impairment are regarded as the risk factors. The manifestation consists of long asymptomatic periods, acute flares with pain and swelling of involved joints, and chronic gouty arthritis with tophi^4^. Uric acid lowering strategy is essential to prevent acute flares and permanent joint damage in gout, and xanthine oxidase inhibitors—allopurinol and febuxostat—are commonly used^5^.

These agents block xanthine oxidases that catalyze the oxidation of hypoxanthine to xanthine and xanthine to uric acid, thereby effectively reducing uric acid synthesis^6^. These hypouricemic agents are used in increasing doses to achieve the target uric acid level of <6.0 mg/dL (360 µmol/L); the American College of Rheumatology (ACR) and European League Against Rheumatism (EULAR) recommendations for the treatment of gout suggest lowering uric acid levels below 5.0 mg/dL (300 µmol/L) in more severe disease conditions with tophi, frequent flares, or chronic gouty arthritis^7,8^.

Gout is well known to be associated with other metabolic disorders such as diabetes, dyslipidemia, or obesity, and especially dyslipidemia with elevated low-density lipoprotein (LDL) cholesterol and hypertriglyceidemia^9^. Elevated serum uric acid contributes to high LDL cholesterol and hypertriglyceridemia, but allopurinol can reduce triglyceride accumulation by decreasing intracellular uric acid levels^10^. Hypertriglyceridemia is caused by alcohol consumption, obesity, and sedentary lifestyle, and has been found to contribute to the development of gout^11,12^. Fenofibrate is the treatment of choice in patients with moderate to severe triglyceridemia because it enhances the oxidation of fatty acids in the liver and muscle and reduces hepatic lipogenesis^13,14^. Fibrates are fibric acid derivatives that activate the nuclear transcription factor peroxisome proliferator-activated receptor (PPAR)-α, which controls fatty acid transport and β-oxidation and results in a reduction in triglyceride levels and an increase in high-density lipoprotein cholesterol levels^15,16^. Fenofibrate is widely used to modify the lipid profile of patients with hypertriglyceridemia or type 2 diabetes and has a preventive effect on the progression of atherosclerosis in those patients^17,18^.

In clinical practice, physicians have prescribed fenofibrate for the treatment of hypertriglyceridemia in patients with gout. Fenofibrate has been reported to reduce serum uric acid levels in other populations^19^. However, the effect on the uric acid level or the adverse effect of fenofibrate coadministered with xanthine oxidase inhibitors has rarely been investigated in patients with gout. Only a few studies have examined by how much the uric acid level could be reduced by adding fenofibrate to patients with gout who received standard therapy.

Therefore, we sought to determine the effect of fenofibrate on the serum uric acid level in patients with gout by comparing serial serum uric acid levels between patients receiving xanthine oxidase inhibitors and fenofibrate and those receiving only xanthine oxidase inhibitors. In addition, we investigated whether fenofibrate coadministration affects renal or hepatic function in these patients.

## Results

### Basic characteristics of patients with gout

Table 1 shows the comparison of characteristics between patients administered allopurinol or febuxostat and patients cotreated with fenofibrate. Of the 863 patients with gout, 70 (8.1%) and 793 (91.9%) had prescriptions for allopurinol plus fenofibrate or febuxostat plus fenofibrate and allopurinol or febuxostat, respectively. Among them, 789 patients (91.4%) were men; the mean age was 50.6 ± 14.9 years; and 394 (45.7%) and 127 (14.7%) patients had hypertension and diabetes, respectively. The patients with gout on allopurinol plus fenofibrate or febuxostat plus fenofibrate were younger (46.9 ± 12.0 vs. 50.9 ± 15.1 years, *p* = 0.012) and had less hypertension (22/70, 31.4 vs. 372/793, 46.9%, *p* = 0.013) than those with gout who were not on fenofibrate. The levels of serum triglyceride decreased from 418.4 ± 190.0 to 316.2 ± 185.2 mg/dL, *p* = 0.002), suggesting good fenofibrate treatment adherence of the patients.

**Table 1:** Characteristics of study participants by treatment groups.

	Total		Allopurinol or febuxostat		Allopurinol plus fenofibrate or febuxostat plus fenofibrate		*P*-value
	N	%	n	%	n	%	
Total	863		793		70		
Sex							0.026
Male	789	91.4	720	90.8	69	98.6	
Female	74	8.6	73	9.2	1	1.4	
Age, years							0.014
–29	53	6.1	51	6.4	2	2.9	
30–39	171	19.8	148	18.7	23	32.9	
40–49	208	24.1	190	24.0	18	25.7	
50–59	194	22.5	180	22.7	14	20.0	
60–69	124	14.4	113	14.2	11	15.7	
70–	113	13.1	111	14.0	2	2.9	
Age (mean/SD)	50.6	14.9	50.9	15.1	46.9	12.0	0.012
Hypertension							0.013
No	469	54.3	421	53.1	48	68.6	
Yes	394	45.7	372	46.9	22	31.4	
Hyperlipidemia							<0.0001
No	563	65.2	554	69.9	9	12.9	
Yes	300	34.8	239	30.1	61	87.1	
Diabetes mellitus							0.806
No	736	85.3	677	85.4	59	84.3	
Yes	127	14.7	116	14.6	11	15.7	
Colchicine							0.683
No	661	76.6	606	76.4	55	78.6	
Yes	202	23.4	187	23.6	15	21.4	
Glucocorticoids							0.247
No	627	72.7	572	72.1	55	78.6	
Yes	236	27.3	221	27.9	15	21.4	
NSAIDs							0.156
No	628	72.8	572	72.1	56	80.0	
Yes	235	27.2	221	27.9	14	20.0	
HMG-CoA reductase inhibitor							0.780
No	820	95.0	753	95.0	67	95.7	
Yes	43	5.0	40	5.0	3	4.3	
Angiotensin receptor							0.424
No	812	94.1	744	93.8	68	97.1	
Yes	51	5.9	49	6.2	2	2.9	
Year of diagnosis							0.904
1995–2000	11	1.3	10	1.3	1	1.4	
2001–2005	124	14.4	114	14.4	10	14.3	
2006–2010	215	24.9	195	24.6	20	28.6	
2011–2015	343	39.7	315	39.7	28	40.0	
2016–2018	170	19.7	159	20.1	11	15.7	

SD: standard deviation; NSAIDs: nonsteroidal anti-inflammatory drugs; HMG-CoA: 3-hydroxy-3-methylglutaryl-CoA.

### Comparison of uric acid reduction between patients treated with and without fenofibrate

**Figure 1:**
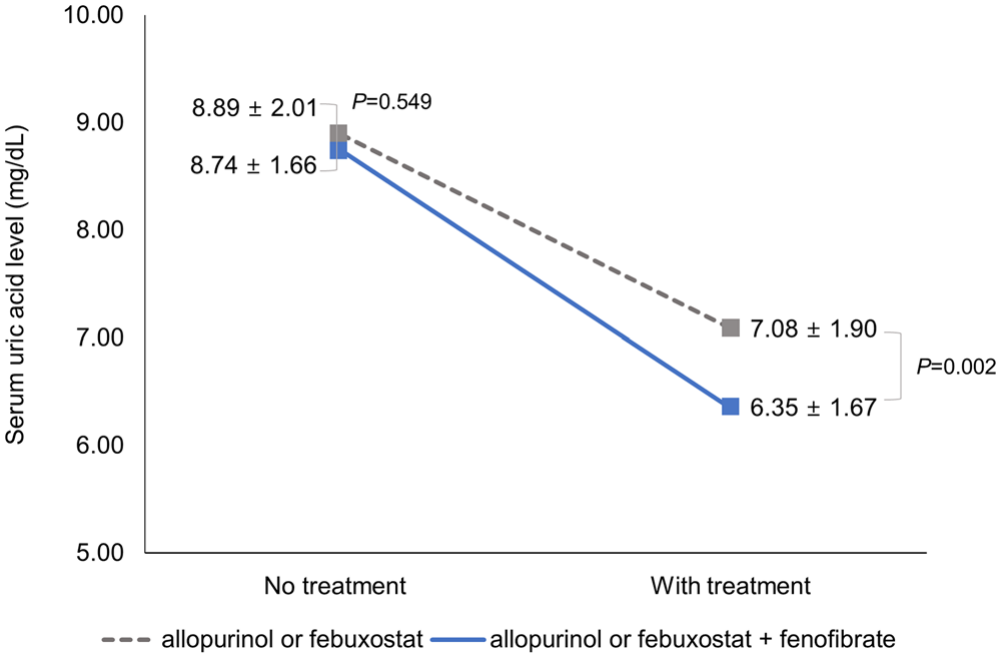
Change in serum uric acid levels according to treatment groups (group without fenofibrate vs. with fenofibrate). Gray dotted lines indicate the reduced levels of uric acid in patients administered allopurinol or febuxostat; blue line indicates the reduced levels of uric acid in the patients co-administered with fenofibrate. The interaction effect on the uric acid levels between treatment groups and whether with or without treatment was not statistically significant (p = 0.084).

Changes in serum uric acid levels with and without fenofibrate are shown in Fig. 1. Before the medication, the average uric acid level did not differ between the patients on allopurinol or febuxostat and those on allopurinol plus fenofibrate or febuxostat plus fenofibrate (8.89 ± 2.01 vs. 8.74 ± 1.66 mg/dL, *p* = 0.548). After treatment, both groups showed decreased serum uric acid levels, which more significantly decreased in patients cotreated with fenofibrate than in those on allopurinol or febuxostat alone (−1.81 ± 2.41 vs. −2.40 ± 2.28 mg/dL, *p* = 0.043).

Table 2 shows the results of the multiple linear regression, which assessed the association between the types of medication and change in serum uric acid levels. Even after adjusting for the confounders, we found that the serum uric acid level decreased more in patients on allopurinol plus fenofibrate or febuxostat plus fenofibrate than it did in patients with gout but not treated with fenofibrate (b = −0.879, *p* = 0.003). Furthermore, serum uric acid levels decreased more in patients with gout who were administered glucocorticoids than in patients who were not (b = −1.098, *p* < 0.001).

**Table 2:** Association between treatment groups (with and without fenofibrate) and change in serum acid level.

Variables	b	S.E.	95% CI		p-value
Treatment groups					
allopurinol or febuxostat	Ref.				
allopurinol or febuxostat + fenofibrate	**−0.879**	**0.300**	**−1.468**	−**0.290**	**0.003**
Sex					
Men	Ref.				
Women	0.456	0.285	−0.103	1.015	0.110
Age					
−29	Ref.				
30–39	0.353	0.353	−0.338	1.044	0.317
40–49	0.213	0.345	−0.464	0.890	0.538
50–59	0.052	0.352	−0.637	0.741	0.883
60–69	−0.105	0.374	−0.837	0.628	0.779
70–	−0.364	0.387	−1.122	0.394	0.346
Hypertension					
No	Ref.				
Yes	−0.330	0.176	−0.675	0.015	0.061
Hyperlipidemia					
No	Ref.				
Yes	0.024	0.180	−0.329	0.376	0.896
Diabetes mellitus					
No	Ref.				
Yes	−0.048	0.230	−0.499	0.404	0.836
Colchicine					
No	Ref.				
Yes	0.158	0.188	−0.211	0.527	0.400
Steroid					
No	Ref.				
Yes	**−1.098**	**0.182**	**−1.454**	**−0.742**	**<0.0001**
NSAID					
No	Ref.				
Yes	0.127	0.177	−0.220	0.475	0.472
HMG-CoA reductase inhibitor					
No	Ref.				
Yes	−0.405	0.388	−1.167	0.356	0.297
Angiotensin receptor					
No	Ref.				
Yes	0.466	0.360	−0.240	1.172	0.195
Year of diagnosis					
1995–2000	Ref.				
2001–2005	0.843	0.701	−0.531	2.218	0.229
2006–2010	1.489	0.692	0.133	2.846	0.031
2011–2015	0.505	0.688	−0.843	1.852	0.463
2016–2018	0.128	0.697	−1.238	1.495	0.854

SD: standard deviation; NSAIDs: nonsteroidal anti-inflammatory drugs; HMG-CoA: 3-hydroxy-3-methylglutaryl-CoA. Outcome variable was change in serum uric acid level (‘after drug administration’ minus ‘before drug administration’). The negative estimation means decreased average serum uric acid level compared with the reference group.

The levels of serum uric acid at enrolment were compared to the levels before the index measure to exclude spontaneous changes of serum uric acid. Pre-enrollment levels of serum uric acid were collected at the closest measurement between 30 and 150 days prior to the time of enrolment. There was no difference in the levels of serum uric acid between before and at enrolment (Supplementary Table 1).

### Comparison of the results of renal and liver function tests between patients treated with and without fenofibrate

Table 3 shows the results of the liver and renal function tests before and after the use of medication according to the treatment groups. Mean creatinine, blood urea nitrogen (BUN), aspartate aminotransferase (AST), and alanine aminotransferase (ALT) levels before and after medication were not significantly different between patients on allopurinol or febuxostat and those on allopurinol plus fenofibrate or febuxostat plus fenofibrate.

**Table 3:** Results of laboratory tests before and after medication use according to medication treatment groups.

	Total N	No treatment		With treatment	
		Mean/n	SD/Row/%	Mean/n	SD/Row%
Creatinine (mg/dL)					
allopurinol or febuxostat	762	1.81	2.01	1.84	2.18
allopurinol or febuxostat plus fenofibrate	68	1.21	0.29	1.26	0.27
BUN (mg/dL)					
allopurinol or febuxostat	760	22.23	17.22	23.07	18.6
allopurinol or febuxostat plus fenofibrate	68	15.54	8.04	15.24	5.07
AST (IU/L)					
allopurinol or febuxostat	760	28.89	22.33	30.15	21.31
allopurinol or febuxostat plus fenofibrate	69	33.58	22.14	33.09	17.6
ALT (U/L)					
allopurinol or febuxostat	759	35.79	44.0	35.72	30.15
allopurinol or febuxostat plus fenofibrate	67	46.16	39.86	40.09	27.23
AST*					
allopurinol or febuxostat	698	11	1.6	16	2.3
allopurinol or febuxostat plus fenofibrate	67	3	4.5	1	1.5
ALT*					
allopurinol or febuxostat	698	26	3.7	31	4.4
allopurinol or febuxostat plus fenofibrate	67	6	9.0	3	4.5
Bilirubin					
allopurinol or febuxostat	755	0.73	0.37	0.76	0.41
allopurinol or febuxostat plus fenofibrate	68	0.71	0.35	0.68	0.25
Total cholesterol					
allopurinol or febuxostat	758	181.0	42.0	180.96	39.83
allopurinol or febuxostat plus fenofibrate	68	203.2	43.7	190.81	38.15
HDL cholesterol					
allopurinol or febuxostat	430	42.91	12.18	42.91	12.18
allopurinol or febuxostat plus fenofibrate	56	39.13	9.99	39.13	9.99
LDL cholesterol					
allopurinol or febuxostat	187	113.71	38.41	109.49	34.26
allopurinol or febuxostat plus fenofibrate	23	120.04	39.71	102.27	42.5
Triglyceride					
allopurinol or febuxostat	469	196.41	106.76	187.71	109.39
allopurinol or febuxostat plus fenofibrate	62	418.42	190.01	316.17	185.15
Glucose					
allopurinol or febuxostat	754	110.41	33.86	106.55	28.27
allopurinol or febuxostat plus fenofibrate	68	106.68	27.09	105.39	27.03

SD: standard deviation; BUN: blood urea nitrogen; AST: aspartate aminotransferase; ALT: alanine aminotransferase; HDL: high density lipoprotein; LDL: low density lipoprotein.

*The number and proportion of patients who exceed three times of the maximum normal range before and after medication.

## Discussion

In addition to gout, hyperuricemia also leads to hypertension, diabetes, and dyslipidemia, and has become a public health burden worldwide because of its high prevalence and clinical significance^20^. Although uric acid has a beneficial effect as an oxygen radical scavenger, hyperuricemia correlates closely with cardiovascular risk and all-cause mortality^21,22^. The aging society and decreased human physical activity caused by social development, have increased the incidence of gout with obesity, dyslipidemia, diabetes, hypertension, and hyperuricemia^6^.

In this study, we analyzed the clinical data of 863 patients with gout who were administered xanthine oxidase inhibitors, and fenofibrate decreased the uric acid level by approximately 0.73 mg/dL. Furthermore, cotreatment with fenofibrate and xanthine oxidase inhibitors did not affect the serum levels of the kidney and liver function markers.

Several studies have examined the effect of fenofibrate on uric acid levels in gout. The studies showed that uric acid levels decreased by 19 and 23% after fenofibrate treatment of patients with gout who were on urate-lowering agents^23,24^. However, although the reduction of urate levels was assessed in a small number (10 and 14) of patients with gout, this study collected sufficient clinical data to evaluate the general effect of fenofibrate. Furthermore, in the previous studies, patients treated with only allopurinol were enrolled, whereas our patients were prescribed allopurinol or febuxostat. Febuxostat is considered more effective than allopurinol in achieving target uric acid levels^25,26^.

The uric acid levels were reduced in patients taking xanthine oxidase inhibitors and fenofibrate compared with those taking only xanthine oxidase inhibitors (−1.81 vs. −2.4 mg/dL, *p* = 0.043). Fenofibrate decreases serum uric acid levels by increasing urinary excretion of uric acid by inhibiting the renal organic anion transporter urate transporter 1 (URAT1; solute carrier family 22 member 12, SLC22A12)^19,27^. Moreover, it was shown to raise urinary pH, and acidic urinary pH could be associated with the formation of uric acid stones, which is one of the comorbidities of gout and a common complication of uricosuric agents^28^. Therefore, the addition of fenofibrate is reasonable, considering that agents that increase the renal excretion of uric acid are likely to lead to the development of uric acid stones.

More studies on the effect of fenofibrate on uric acid reduction were carried out in patients with hypertriglyceridemia or diabetes^19,29,30^. In the randomized and controlled fenofibrate intervention and event lowering in diabetes (FIELD) study conducted to determine the effect of fenofibrate on type 2 diabetes, uric acid levels were decreased by 10.3% in 622 patients with diabetes who took allopurinol. In addition, the first gout event in 5 years was more frequent with placebo treatment than with fenofibrate among patients with diabetes^31^.

Treatment strategies for acute gout such as the use of anti-inflammatory drugs including colchicine, nonsteroidal anti-inflammatory drugs (NSAIDs), and glucocorticoids are frequently maintained because of the need for prophylaxis to prevent further recurrent gout flares^5,7^. In this study, among the treatments with anti-inflammatory drugs, statins, and angiotensin receptor modulating agents, glucocorticoids were correlated significantly with uric acid reduction. This finding could be explained by the fact that glucocorticoids enhance urinary excretion of uric acid by directly acting on uric acid transport in the renal tubules^30–32^. Moreover, the effect of glucocorticoids on lowering uric acid was similar to that of allopurinol in previous reports^32–34^.

Since mild liver biochemical abnormalities are known to occur in 5–10% of patients on fenofibrate, baseline and regular periodic aminotransferase testing is recommended^35^. Fenofibrate-induced hepatotoxicity is hepatocellular, cholestatic, or of a mixed pattern, and ranges from an acute and self-limiting to chronic liver injury^36^. However, in this study, there was no differences in the levels of AST and ALT between patients treated with and without fenofibrate. Furthermore, previous studies showed the beneficial effects of fenofibrate on levels of liver enzymes in patients with fatty liver and its preventive effects on fatty liver disease in mice^37,38^. In several studies including a large-scale randomized controlled trial, 2% of the patients taking fenofibrate had more elevated creatinine levels when compared with the control group patients^14,39^. Fenofibrate-associated nephrotoxicity occurs in patients with pre-existing renal disease, and high dosage poses a higher risk than low dosage^40^. However, our patients with gout showed no change in levels of creatinine and BUN before and after treatment, and no difference occurred between patients treated with and without fenofibrate.

A limitation of this study is the lack of data on gout flares, which should ultimately be prevented in the treatment of gout. However, this is a novel study that investigated the effect of fenofibrate on the reduction of uric acid in a large population of patients with gout who were administered febuxostat and allopurinol. The status of these patients is significant because it is similar to that in the current clinical situation, indicating that it could be applied to the management of patients with gout and elevated triglycerides. Another limitation is that during the study period of more than 20 years, there have been changes in the use of the drug for lowering serum uric acid level because febuxostat was introduced in 2012 in the subject hospital. However, there was no significant difference in the ratio of the prescription count of allopurinol and febuxostat between two the treatment groups (group without fenofibrate vs. with fenofibrate) as shown in Supplementary Table 2. Finally, our analysis did not contain a confirmation for patient adherence to medication. It is a well-known and inevitable limitation of retrospective data. However, the level of adherence would be randomly distributed in both treatment groups, and there are no indications or factors that adherence was higher in a certain group as compared to that in the other group.

In conclusion, this data shows that the addition of fenofibrate reduced uric acid levels in patients with gout who were taking allopurinol or febuxostat, and they showed no significant change in renal or hepatic functions. Therefore, cotreatment of fenofibrate with xanthine oxidase inhibitors might be a favorable therapeutic strategy in patients with gout who have hypertriglyceridemia.

## Methods

### Data source and study participants

We used the data from the electronic medical record (EMR) system of the Ajou University Hospital, which has been operating as a tertiary teaching hospital in Korea since 1994. The EMR system collects information based on unique, de-identified numbers for patients combined with their age and sex, the diagnostic codes based on the International Classification of Diseases (ICD-10), admission and discharge dates, laboratory test results, and prescribed medications/treatments from about 2.9 million patients (including 543,617 inpatients). The information about diagnostic codes including combined diseases was obtained from the EMR and validated by manual chart review (H.-A.K. and J.-Y.J).

To investigate the effect of allopurinol or febuxostat with fenofibrate on uric acid levels, we selected 3,608 patients with gout (ICD-10 code: M10.x) between 1995 and 31 February 2018. Among the 3,608 patients, we first selected patients treated for at least 30 consecutive days with allopurinol or febuxostat (n = 2,299). We excluded those who did not have a uric acid test within 2 months before taking allopurinol or febuxostat (n = 710) and within 2 months after taking allopurinol or febuxostat for 30 days (n = 566).

**Figure 2:**
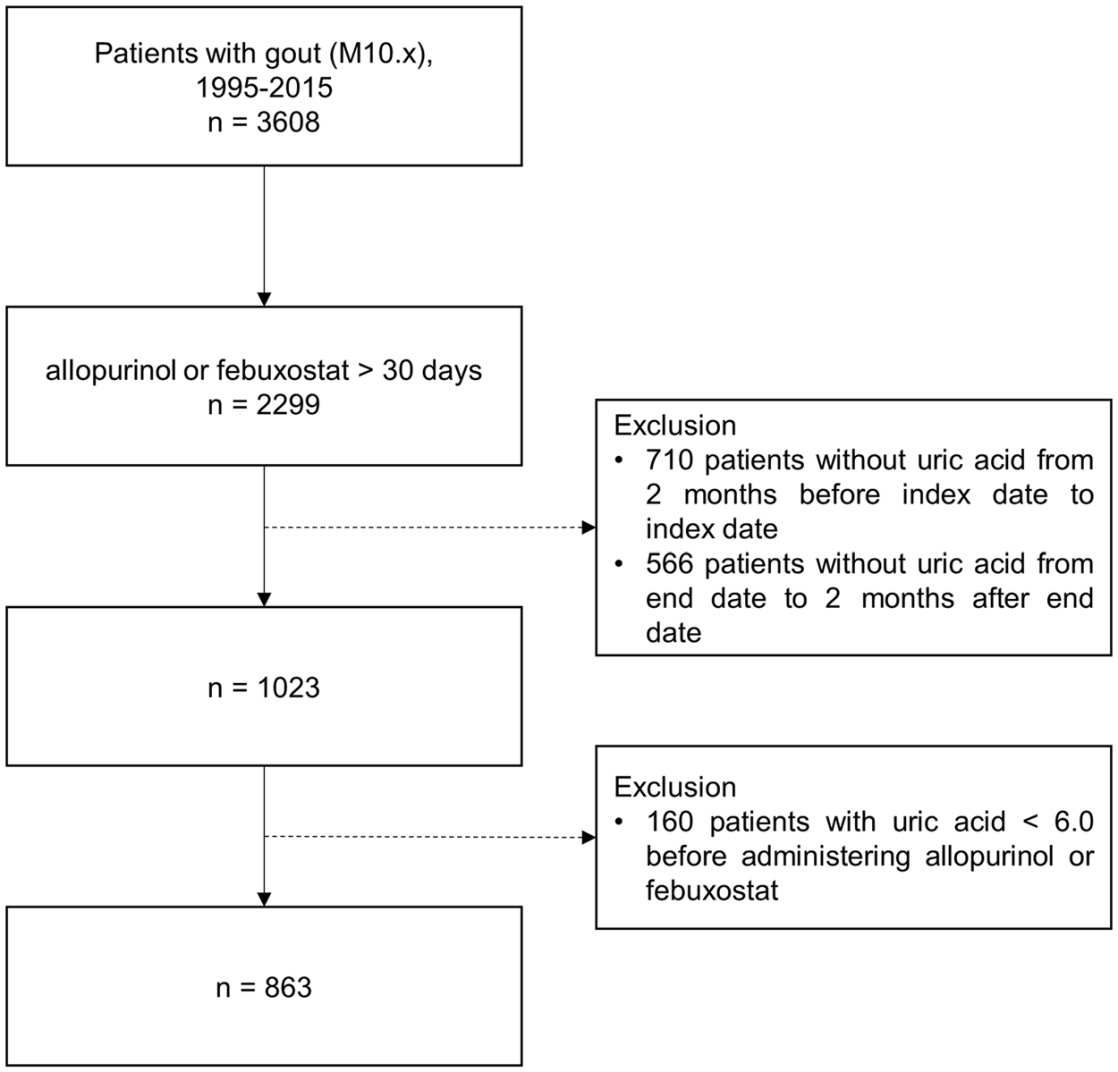
Flowchart depicting the method of selection of the study participants. Data of 1,456 patients were excluded during the collection of laboratory results.

We excluded 160 patients with uric acid levels <6.0 before medication use. Finally, this study included the 863 patients with gout. The flowchart for selecting the study participants is displayed in Fig. 2. In addition, we eliminated 98 patients who did not have renal and liver function tests within 2 months before taking allopurinol or febuxostat and within 2 months after taking allopurinol or febuxostat for 30 days, to examine whether patients taking additional fenofibrate had adverse drug effects on the liver or kidneys.

### Outcomes

The primary outcome was serum uric acid (mg/dL), which was measured within 2 months before the date of prescription of hypouricemic agents and within 2 months after a consecutive 30-days of prescription of hypouricemic agents. Renal (creatinine and BUN) and liver (AST and ALT) function tests were performed within the same period.

### Exposure

The patients were classified into two treatment groups using prescribed medication based on data extracted from the EMR of Ajou University: (1) the group administered allopurinol or febuxostat for 30 consecutive days, and (2) the group co-administered fenofibrate during 30-days of allopurinol or febuxostat treatment.

### Confounders

Data on demographics, comorbidities, and use of other medication were included in this study. All confounders were assessed during the period of exposure. The demographic factors were sex and age. Comorbidities (hypertension, hyperlipidemia, and diabetes mellitus) were included by reviewing the patients’ prescriptions in the medical records. Medication for the treatment of patients with gout were included: NSAIDs, glucocorticoids (methylprednisolone, prednisolone, deflazacort, triamcinolone, and hydrocortisone), and colchicine. This study also included drugs that could affect serum uric acid level, viz., statin and angiotensin receptors.

### Statistical analysis

We compared the characteristics of the allopurinol or febuxostat and allopurinol plus fenofibrate or febuxostat plus fenofibrate groups using chi-square and independent *t*-test. We used the independent *t*-test to compare the difference in serum uric acid levels between the two groups (allopurinol or febuxostat vs. allopurinol plus fenofibrate or febuxostat plus fenofibrate). Two-way ANOVA was used for testing the interaction effect between two the treatment groups and time. Multiple linear regression was conducted to determine the effect of fenofibrate on the difference (‘after drug administration’ minus ‘before drug administration’) in serum uric acid levels in patients with gout. To examine whether patients coadministered fenofibrate had adverse drug reactions, we calculated the average creatinine, BUN, AST, and ALT values before and after medication based on the groups. For the liver function test, we calculated the number and proportion of patients who exceed three times of the maximum normal range before and after medication. A *p* < 0.05 was considered statistically significant. Data were managed using the Microsoft SQL Server (Microsoft Corp), and all statistical analyses were conducted using the SAS software package (ver. 9.4; SAS Institute, Cary, NC, USA).

### Ethics statement

This study was approved by the Institutional Review Board of Ajou University Hospital (AJIRB-MED-OBS-18-102), which waived the requirement for informed consent because the anonymized data were analyzed retrospectively.

## Electronic supplementary material

Supplementary Tables

## Data Availability

The datasets analyzed during the current study are not publicly available due to legal restrictions imposed by the government of South Korea in relation to the Personal Information Protection Act, but are available from the corresponding author on reasonable request.
