# Supplementary material for: Effect of fenofibrate on uric acid level in patients with gout
Source: Sci Rep. 2018 Nov 13;8:16767. doi: 10.1038/s41598-018-35175-z (PMC6233215; doi:10.1038/s41598-018-35175-z)
Supplement: Supplementary file 1 — Supplementary Tables [file 41598_2018_35175_MOESM1_ESM.docx]

**Supplementary Materials**

**Title: Effect of fenofibrate on uric acid level in patients with gout**

Ju-Yang Jung, MD^1^; Young Choi, PhD^2,3^; Chang-Hee Suh, MD, PhD^1^; Dukyong Yoon, MD, PhD^2,3^; and Hyoun-Ah Kim, MD, PhD^1^

^1^Department of Rheumatology, ^2^Department of Biomedical Informatics, ^3^Department of Biomedical Science, Ajou University School of Medicine, 164 Worldcup-ro, Yeongtong-gu, Suwon 16499, Korea

Supplementary Table 1. Difference of uric acid level between before enrolment and the index measure

|  | Total | | Allopurinol or febuxostat | | | | Allopurinol or febuxostat + fenofibrate | |  | |
| --- | --- | --- | --- | --- | --- | --- | --- | --- | --- | --- |
|  | Mean | S.D. | | Mean | S.D. | Mean | | S.D. | | p-value |
| Before | 8.91 | 2.69 | | 8.92 | 2.69 | 8.45 | | 2.89 | | 0.57 |
| At enrollment | 9.16 | 2.40 | | 9.17 | 2.42 | 8.93 | | 2.00 | | 0.74 |
| Difference* | 0.25 | 2.42 | | 0.24 | 2.43 | 0.47 | | 2.29 | | 0.76 |

Supplementary Table 2. Prescription count of allopurinol and febuxostat in the subjects by year

|  | Without fenofibrate | | | | | With fenofibrate | | | | |
| --- | --- | --- | --- | --- | --- | --- | --- | --- | --- | --- |
|  | Total | Allopurinol | | Febuxostat | | Total | Allopurinol | | Febuxostat | |
|  | n | n | % | n | % | n | n | % | n | % |
| 1998 | 24 | 24 | 100.0 | 0 | 0 | 0 | 0 | - | 0 | - |
| 1999 | 92 | 92 | 100.0 | 0 | 0 | 0 | 0 | - | 0 | - |
| 2000 | 83 | 83 | 100.0 | 0 | 0 | 1 | 1 | 100.0 | 0 | 0 |
| 2001 | 128 | 128 | 100.0 | 0 | 0 | 5 | 5 | 100.0 | 0 | 0 |
| 2002 | 164 | 164 | 100.0 | 0 | 0 | 4 | 4 | 100.0 | 0 | 0 |
| 2003 | 233 | 233 | 100.0 | 0 | 0 | 15 | 15 | 100.0 | 0 | 0 |
| 2004 | 294 | 294 | 100.0 | 0 | 0 | 20 | 20 | 100.0 | 0 | 0 |
| 2005 | 374 | 374 | 100.0 | 0 | 0 | 11 | 11 | 100.0 | 0 | 0 |
| 2006 | 449 | 449 | 100.0 | 0 | 0 | 36 | 36 | 100.0 | 0 | 0 |
| 2007 | 484 | 484 | 100.0 | 0 | 0 | 61 | 61 | 100.0 | 0 | 0 |
| 2008 | 570 | 570 | 100.0 | 0 | 0 | 58 | 58 | 100.0 | 0 | 0 |
| 2009 | 645 | 645 | 100.0 | 0 | 0 | 46 | 46 | 100.0 | 0 | 0 |
| 2010 | 721 | 721 | 100.0 | 0 | 0 | 84 | 84 | 100.0 | 0 | 0 |
| 2011 | 796 | 796 | 100.0 | 0 | 0 | 68 | 68 | 100.0 | 0 | 0 |
| 2012 | 732 | 501 | 68.4 | 231 | 31.6 | 73 | 54 | 74.0 | 19 | 26 |
| 2013 | 997 | 436 | 43.7 | 561 | 56.3 | 95 | 52 | 54.7 | 43 | 45.3 |
| 2014 | 1245 | 643 | 51.6 | 602 | 48.4 | 117 | 65 | 55.6 | 52 | 44.4 |
| 2015 | 1214 | 516 | 42.5 | 698 | 57.5 | 147 | 73 | 49.7 | 74 | 50.3 |
| 2016 | 1346 | 487 | 36.2 | 859 | 63.8 | 134 | 55 | 41.0 | 79 | 59.0 |
| 2017 | 1352 | 304 | 22.5 | 1048 | 77.5 | 132 | 30 | 22.7 | 102 | 77.3 |
| 2018 | 118 | 23 | 19.5 | 95 | 80.5 | 7 | 1 | 14.3 | 6 | 85.7 |
| **Total** | 12061 | 7967 | **66.1** | 4094 | **33.9** | 1114 | 739 | **66.3** | 375 | **33.7** |
